# Bacterial dysentery complicated with Crohn’s disease: A case report

**DOI:** 10.1097/MD.0000000000043984

**Published:** 2025-08-15

**Authors:** Xi Wang, Wen Ming, Hui Chen, Jingtong Tang, Guobin He

**Affiliations:** a Department of Gastroenterology, Affiliated Hospital of North Sichuan Medical College, Nanchong, Sichuan Province, China.

**Keywords:** bacterial dysentery, computed tomography enterography, Crohn’s disease, enteroscopy, pathological examination

## Abstract

**Rationale:**

Crohn’s disease is easily confused with bacillary dysentery in clinical manifestations. Crohn’s disease is a chronic disease that can involve both intestinal and extraintestinal areas, and severe patients have prolonged and poor prognosis, so early diagnosis and treatment are crucial for patients. This case is a combination of bacillary dysentery and Crohn’s disease, which emphasizes the differentiation between bacillary dysentery and Crohn’s disease, and expects clinicians to pay attention to and discover the early manifestations of Crohn’s disease.

**Patient concerns:**

A young male was admitted to the hospital with recurrent abdominal pain. Based on the patient’s symptoms, imaging, enteroscopy, and pathology, we concluded that he had “acute bacillary dysentery and Crohn’s disease combined with bladder fistula.”

**Diagnoses:**

1. Acute bacillary dysentery, 2. Crohn’s disease combined with bladder fistula, 3. urinary tract infection.

**Interventions:**

The patient was treated with 390 mg intravenous infusion of ustekinumab, anti-infection, antispasmodic, regulation of intestinal flora, repair of intestinal mucosa, fluid rehydration, nutritional support, etc.

**Outcomes:**

The patient’s abdominal pain was improved, no more blood in the stool, urinary frequency and pain were improved, and urine color was clear.

**Lessons:**

In the course of diagnosis and treatment, the patient’s bacillary dysentery was clearly diagnosed, so the corresponding treatment was given, and there was a good effect in the treatment process, so the possibility of complications was not paid attention to. At the same time, Crohn’s disease (CD) is a chronic nonspecific inflammation of the intestine, which is difficult to diagnose. Moreover, some manifestations of CD under endoscopy are similar to those of bacillary dysentery, which increases the difficulty of diagnosis. In this case, the initial treatment of bacillary dysentery was good, but CD was found in the later stage when the disease was repeated and complications occurred, delaying the diagnosis and treatment of CD. Therefore, this paper reviewed part of the literature, summarized and identified the epidemiology, clinical manifestations, endoscopic manifestations, and pathological manifestations of bacillary dysentery and CD, hoping that clinicians can pay attention to and discover the early manifestations of CD.

## 1. Introduction

Crohn’s disease (CD) and bacillary dysentery are easily confused in clinical manifestations, especially in acute episodes or atypical cases, both of them may present similar symptoms such as abdominal pain, diarrhea, fever, etc. When CD involves the colon, the manifestations of mucous, pus and blood stool may overlap with those of bacillary dysentery. In addition, if bacillary dysentery is left untreated or becomes a chronic infection, it may be confused with the chronic course of CD due to persistent diarrhea and intestinal inflammation.

## 2. Case report

**Figure 1.**
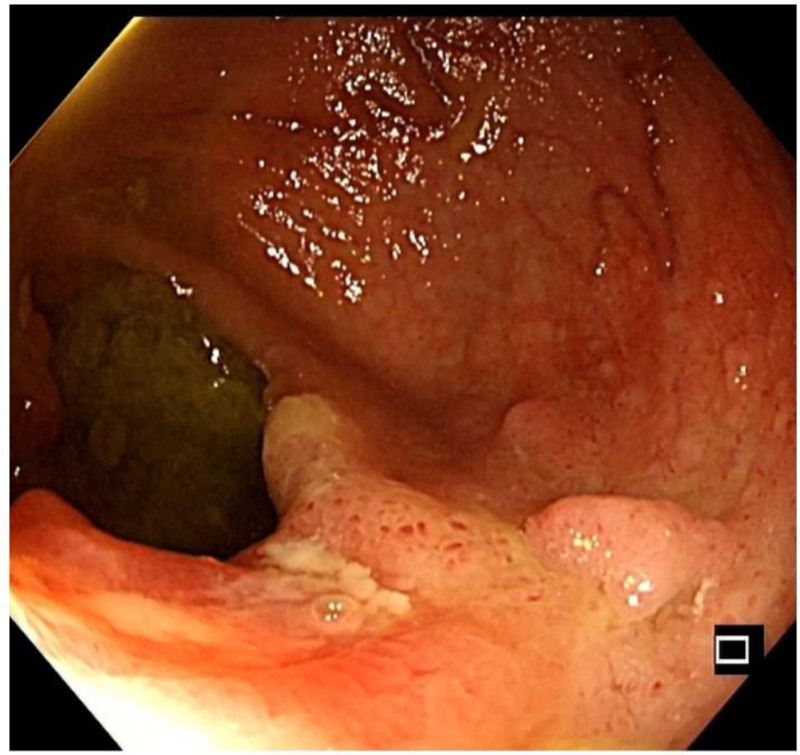
Enteroscopic manifestations in the diagnosis of bacillary dysentery.

**Figure 2.**
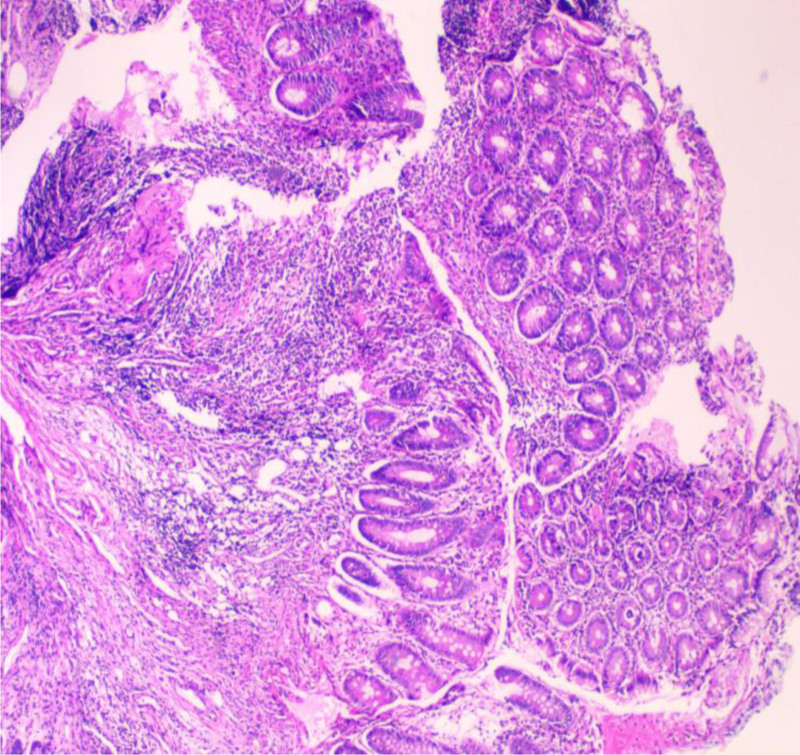
Pathologic manifestations in the diagnosis of bacillary dysentery (hematoxylin and eosin staining × 100).

A young male with a height of 173.0 cm and a weight of 55.0 kg was admitted to hospital for abdominal pain for >2 months, mainly manifested as periumbilical paroxysmal distension and pain, which was obvious after eating and could be relieved after defecation, accompanied by mucous, abscess and blood stool, about 1 to 2 times a day, accompanied by rigiditis and vomiting, without fear of cold, fever, nausea and vomiting. The patient bought berberine, probiotics, and montmorillonite powder on his own outside the hospital, which had poor results, and he came to our hospital for further diagnosis and treatment. Since the onset of the disease, the patient’s spirit, appetite, and sleep were normal, stool were as described above, and urination occasionally had a burning sensation. He had lost about 2 kg since his illness. His past history, personal history and family history were unremarkable. At the time of admission, he was found to have scattered pressure pain in the whole abdomen, especially around the umbilicus, with obvious rebound pain, accompanied by abdominal muscle tension, active bowel sounds, and no positive signs on the rest of the examination. Blood routine showed white blood cell 10.08 × 10^9^/L, hemoglobin 112 g/L. C-reactive protein 72.30 mg/L. Interleukin-6 19.57 pg/mL. Procalcitonin < 0.05 ng/mL. Urine routine white blood cell count 28/uL. Fecal occult blood was positive, red cells 1 to 2, white cells 0 to 1. Fecal smear showed normal total bacterial count, yeast-like fungi were detected, gram-negative bacteria 96%, gram-positive bacteria 1%, yeast like fungi 3%. Shigella fowleri was found in fecal culture. Abdominal ultrasound showed “1. Urinary salt deposition in the bladder. 2. A small amount of fluid accumulation in the pelvic cavity.” Enteroscopy showed “longitudinal and transverse ulcers in the terminal ileum, nodular ileocecal valve, and no abnormality in the orifice of vermiform appendix. The mucosa of the sigmoid colon showed nodular hyperplasia with surface erosion, and the mucosa of the rectum was smooth,” see Fig. 1. Pathological examination revealed “severe chronic inflammation of the mucosa of the terminal ileum and sigmoid colon, with a diffuse infiltration of acute and chronic inflammatory cells in the lamina propria,” see Fig. 2. The patient was comprehensively diagnosed as “acute bacillary dysentery,” and was treated with anti-infection, antispasmodic, regulation of intestinal flora, repair of intestinal mucosa, rehydration, nutritional support, etc. The patient’s abdominal pain improved, and he was not relieved of mucus, pus, and blood, and was discharged from the hospital with improved condition.

**Figure 3.**
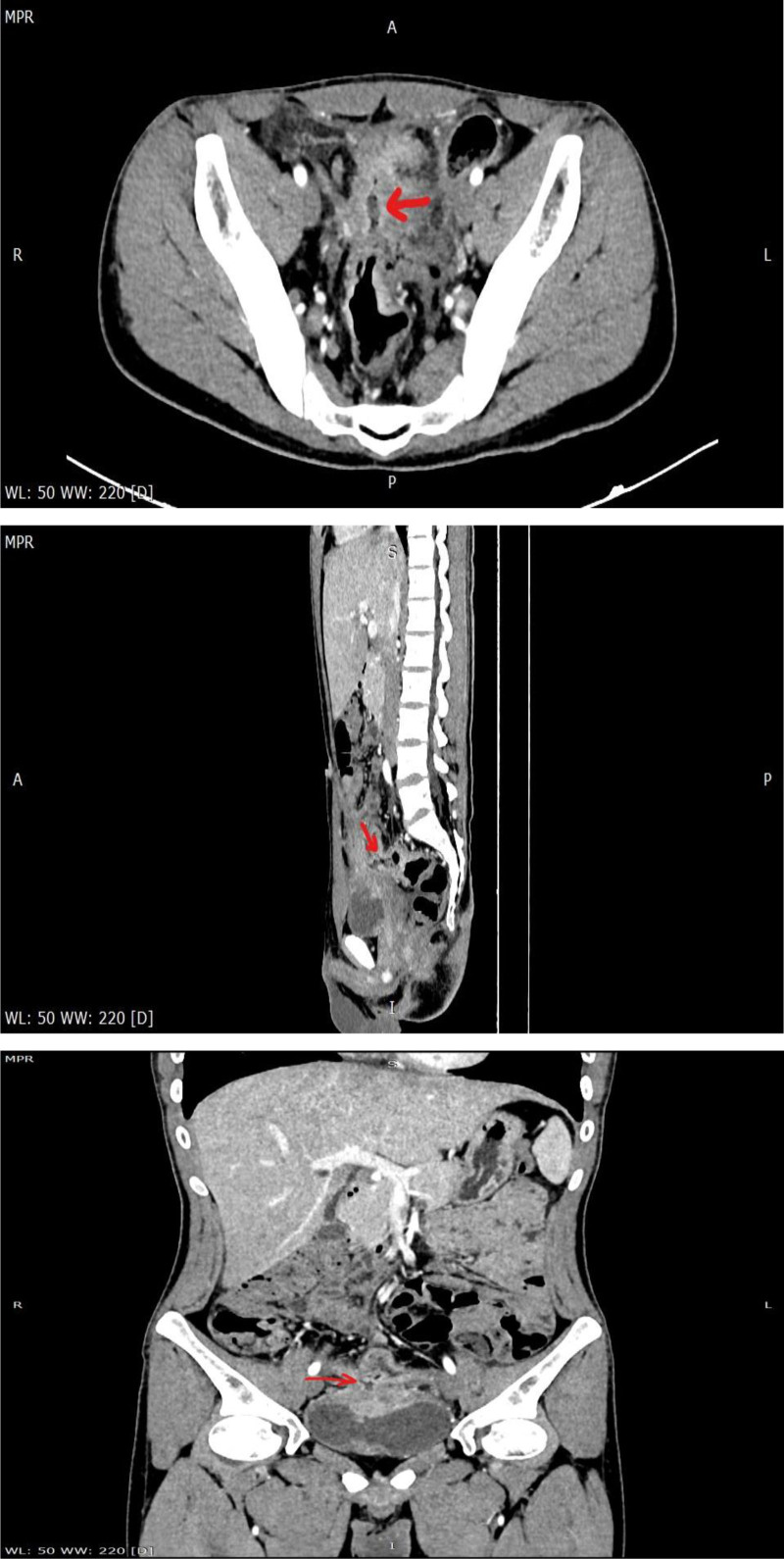
CT manifestations of the small bowel in the diagnosis of Crohn’s disease combined with bladder fistula. CT = computed tomography.

**Figure 4.**
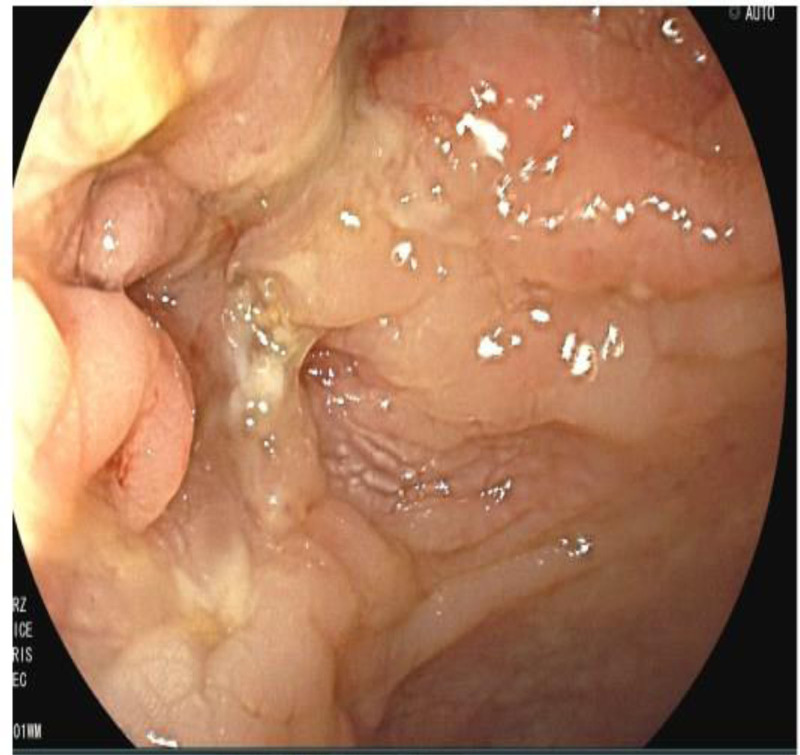
Enteroscopic manifestations in the diagnosis of Crohn’s disease combined with bladder fistula.

**Figure 5.**
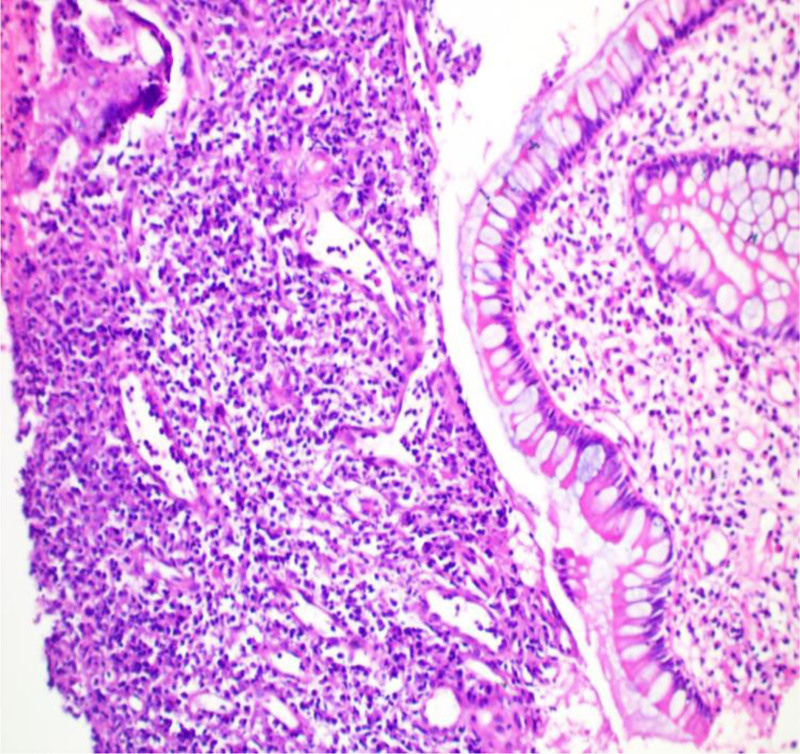
Pathologic manifestations in the diagnosis of Crohn’s disease combined with bladder fistula (hematoxylin and eosin staining × 100).

One month after discharge, the patient had abdominal pain again, including periumbilical paroxysmal distending pain accompanied by blood in stool, mostly blood on stool surface, small amount, 1 to 2 times a day, occasional mucous blood stool, accompanied by posterior tenesia, accompanied by frequent urination, pain in urine, and occasionally cloudy urine. The patient was diagnosed as “urinary tract infection” at a local hospital, and no significant relief of symptoms was observed after oral treatment with amoxicillin and clavulanate potassium tablets. The patient visited our hospital again for further diagnosis and treatment. On admission, the right lower abdomen tenderness and rebound pain were examined, and no positive signs were found in the remaining physical examination. Fecal routine occult blood immunoassay was positive. Fecal smear showed yeast like fungi. *Candida albicans* was found in fecal culture. Urine routine white blood cell count 2597/uL, nitrite +, occult blood 3+, leukocyte esterase 500 Leu/uL, red blood cell count 229/uL. Gram-negative bacilli and gram-positive cocci were found in urine smear. *Escherichia coli* and *Enterococcus faecium* were detected in urine culture. Blood routine showed white blood cell 6.65 × 10^9^/L, erythrocyte sedimentation rate analysis 63 mm/h, whole blood hypersensitive C-reactive protein 24.37 mg/L, platelets 454 × 10^9^/L, hemoglobin 100 g/L. Liver and renal function, electrolytes, cardiac enzymes, carcinoembryonic antigen, herpes simplex virus antibodies, EBV DNA, cytomegalovirus DNA, pre-transfusion immunization, connective tissue-associated antibodies, tuberculosis-infected T-cell gamma-interferon release assay, and anti-neutrophil plasma antibodies did not show any significant abnormality. The purified protein derivative test was negative. Chest computed tomography (CT) low-dose scan of the heart and lungs did not show any definite abnormality. Appendiceal ultrasound showed no definite abnormality. Male urologic ultrasound suggested “1. Bladder considered focal inflammatory changes. 2. Urinary salt deposited in the bladder.” Transrectal perianal ultrasound showed no significant abnormalities. Computed tomography enterography showed “1. Thickening of the intestinal wall of the right lower abdominal ileum, ileocecal part and sigmoid colon, blurring of the surrounding fat space, enhanced scanning showed obvious enhancement, the boundary between the diseased segment of sigmoid colon and the adjacent small intestine was unclear, local irregular mass changes were observed, and the boundary between the diseased segment and the adjacent upper bladder wall was unclear, the bladder wall was thickened, and a small amount of gas density shadow was seen in the bladder cavity. Considering infectious lesions involving the bladder. 2. Pelvic mesentery thickened, mesenteric space was slightly blurred, multiple lymph node shadows were seen, and some parts were slightly enlarged. 3. A small amount of fluid in the pelvic cavity,” see Fig. 3. Gastroscopy showed “chronic non-atrophic gastro-sinusitis.” Enteroscopy showed “nodular neoplasm in the ileocecal valve and part of the ascending colon, with no obvious ileocecal valve orifice, and luminal stenosis, nodular neoplasm, and ulcerative changes in part of the colon at about 20 to 10 cm from the anus. The mucosa of the remaining colon was smooth,” see Fig. 4. Pathological examination showed “chronic inflammation of the mucosa of the terminal ileum and sigmoid colon with erosions, moderate lymphocytic infiltration of the lamina propria, a small number of neutrophils, a slight proliferation of granulomatous tissue, no granulomatous structures, no coarse, branching crypts, and no fissure ulcers. The ileum, ascending colon, transverse colon, descending colon, and rectum all showed mild chronic inflammation of the mucosa. CMV (‐), EBER (‐), and antacid (‐),” see Fig. 5. After comprehensive consideration, the patient was diagnosed as “1. Crohn’s disease combined with bladder fistula 2. Urinary tract infection,” and was treated with 390 mg intravenous infusion of ustekinumab, anti-infection, regulation of intestinal flora, repair of intestinal mucosa, fluid rehydration, nutritional support, etc. The patient’s abdominal pain was improved, no more blood in the stool, urinary frequency and pain were improved, and urine color was clear.

**Figure 6.**
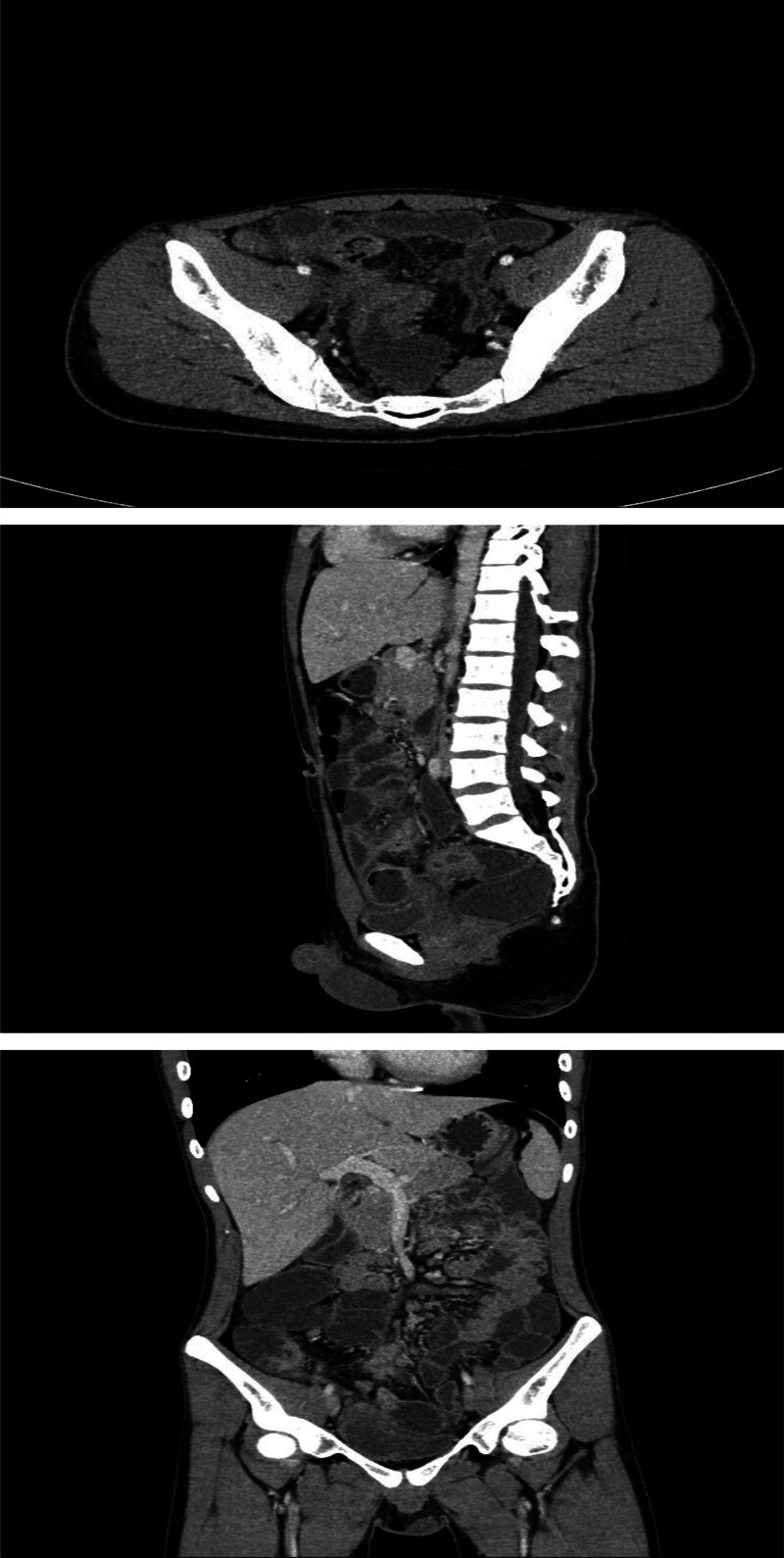
CT manifestations of the small bowel on review after treatment of Crohn’s disease combined with bladder fistula. CT = computed tomography.

**Figure 7.**
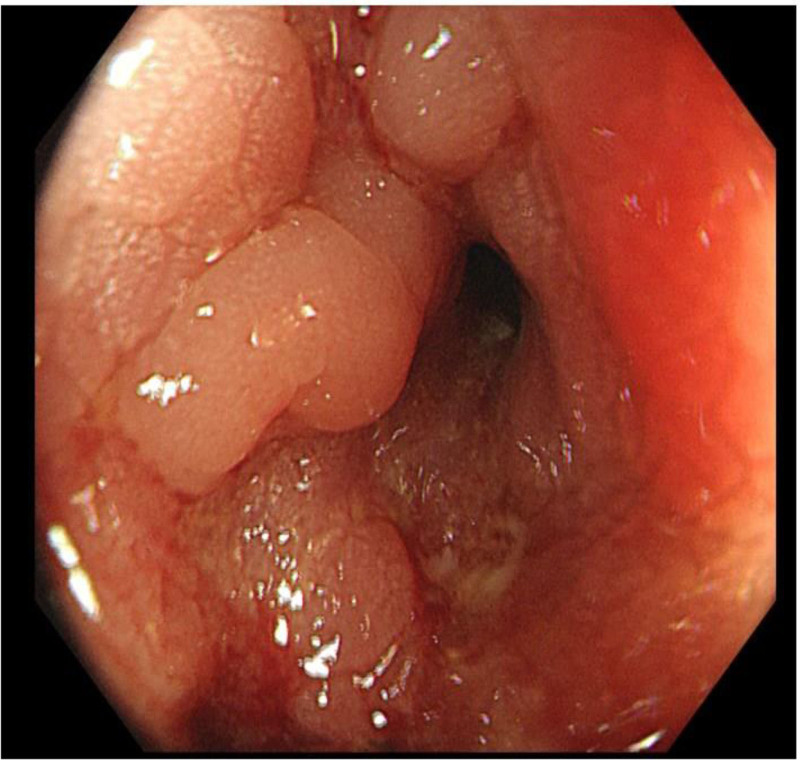
Enteroscopic manifestations on review after treatment of Crohn’s disease combined with bladder fistula.

**Figure 8.**
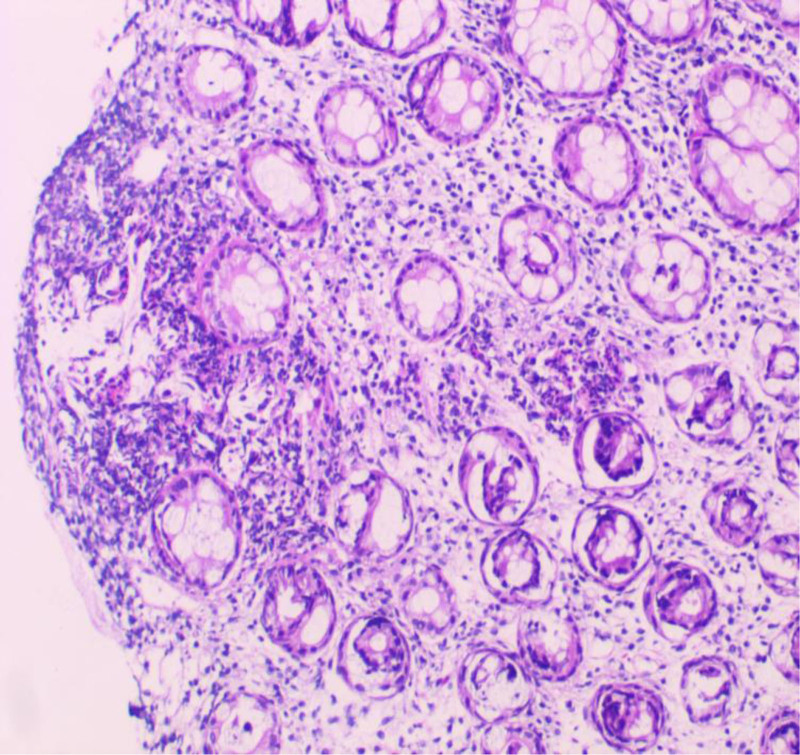
Pathologic manifestations on review after treatment of Crohn’s disease combined with bladder fistula (hematoxylin and eosin staining × 100).

Reexamination of urine routine showed white blood cell count 480/uL, leukocyte esterase 500 Leu/uL, red blood cell count 130/uL. Computed tomography enterography showed “1. The above changes in the distal ileum of the right lower abdomen, ileocecal intestine wall, sigmoid colon, and bladder were accompanied by pelvic mesangial thickening and slightly enlarged mesenteric lymph nodes. Considering that infectious lesions involved multiple organs in the pelvic cavity, compared with abdominal pelvic CT images from previous, the thickening degree of small intestine was less than before, and the gas in the bladder cavity was more absorbed than before. 2. A small amount of pelvic fluid was basically absorbed,” see Fig. 6. Enteroscopy showed “the entry reached the transverse colon 50 cm from the anus, and the entry resistance was felt to stop. In the 15–18 cm from the anus, there was significant congestion and swelling of the nodular mucosa, which was easy to bleed when touched, and the biopsy was soft, resulting in narrowing of the intestinal lumen, so the colonoscope could still be passed, but the rest of the colonic mucosa was smooth, with a clear vascular texture and a regular colonic pouch, and there were no ulcers or neoplastic organisms in the lumen. Rectal mucosa was smooth with scattered congestion,” see Fig. 7. Pathological examination showed “chronic inflammation of intestinal mucosa 15 to 18 cm from the anus with edema, diffuse infiltration of medium amount of lymphocytes and plasma cells, and scattered neutrophils in the infiltration,” see Fig. 8. After comprehensive evaluation, the patient’s condition improved, the treatment was effective at present, and ustekinumab treatment was continued regularly in the later stage.

## 3. Discussion

Bacterial dysentery is an intestinal infectious disease caused by Shigella,^[1]^ mainly transmitted through the digestive tract. The main manifestations of bacillary dysentery are abdominal pain, diarrhea, mucous, pus and blood stools, as well as a sense of urgency and heaviness, which may be accompanied by fever and systemic toxemia, and in severe cases, infectious shock and/or toxic encephalopathy may occur.

The pathological changes of bacillary dysentery mainly occur in the large intestine, including inflammation and ulceration of the rectum and sigmoid colon,^[2]^ and in severe cases, the entire colon and the end of the ileum. The typical course of acute bacillary dysentery consists of an initial acute cicatrization, followed by characteristic pseudomembranous inflammation and ulceration, and finally healing. The basic pathological changes in the intestinal mucosa are diffuse fibrin exudative inflammation, early visible punctate hemorrhage, further development of the lesion, intestinal mucosal epithelium formation of superficial necrosis, and a large amount of mucopurulent exudate on the surface. The exudate has a large amount of cellulose, which together with necrotic tissue, inflammatory cells, erythrocytes and bacteria form a characteristic pseudomembrane. About 1 week, the pseudomembrane begins to fall off, forming “map-like” ulcers of different sizes and shapes. However, it is limited to the submucosa, so intestinal perforation and intestinal bleeding are rare. Chronic bacillary dysentery intestinal mucosal edema and thickening of the intestinal wall, ongoing formation and repair of intestinal mucosal ulcers, leading to scarring and polyp formation, and in a few cases narrowing of the intestinal lumen.

The diagnosis of bacillary dysentery is usually based on a combination of epidemiological history, signs and symptoms, and laboratory examination, and the definitive diagnosis depends on etiological examination. Bacillary dysentery occurs most often in summer and autumn,^[3]^ with a history of unclean diet or contact with patients with bacillary dysentery. Clinical manifestations of the acute phase of bacillary dysentery are fever, abdominal pain, diarrhea, urgency and mucous, pus and blood stools, and obvious tenderness in the left lower abdomen. Patients with chronic bacillary dysentery have a history of acute bacillary dysentery and have been ill for more than 2 months without recovery. The diagnosis of bacillary dysentery is made by fecal microscopy with large numbers of leukocytes or pus cells ≥ 15/higher field of view (400 times), erythrocytes and phagocytes. Confirmation of the diagnosis depends on fecal culture for dysentery bacilli.^[2]^

CD is a chronic inflammatory granulomatous disease that occurs mostly in the terminal ileum and adjacent colon, but can affect all sections of the digestive tract from the mouth to the anus in a segmental or jumping distribution. Clinically, it is characterized by abdominal pain, diarrhea, weight loss, abdominal mass, fistula formation and intestinal obstruction, which may be accompanied by systemic manifestations such as fever and extraintestinal damages to the joints, skin, eyes and oral mucosa. Severe cases are prolonged and have a poor prognosis.

The general morphologic features of CD are as follows: (1) the lesions are segmental or jumping, not continuous; (2) the mucosal ulcers appear as thrush-like ulcers in the early stage; then the ulcers increase in size, fuse, and form longitudinal ulcers and fissure ulcers, which divide the mucosa in a cobblestone-like appearance; (3) the lesions involve the whole layer of the intestinal wall, with the wall thickening and hardening, and the lumen of the intestines narrowing. The histologic features of CD are as follows^[4]^: (1) non-caseous granulomas, composed of epithelioid cells and multinucleated giant cells, which can occur in all layers of the intestinal wall and in localized lymph nodes; (2) fissure ulcers, in the shape of a slit, can reach the submucosa or even muscle layer; (3) inflammation in all layers of the intestinal wall, accompanied by aggregation of lymphocytes at the base of the lamina propria and submucosal layer, widening of the submucosal layer, dilatation of the lymphatic vessels, and ganglitis. Intestinal obstruction may occur due to lumen stenosis caused by lesions of whole-layer intestinal wall. Perforated ulcers cause local abscesses, or penetrate into other intestinal segments, organs, and abdominal walls, forming internal or external fistulas. Fibrinous exudation from the plasma membrane of the intestinal wall and chronic perforation can cause intestinal adhesions. CT examination is now widely used in the diagnosis of intestinal diseases, and is also the imaging basis for identifying intestinal diseases. Segmental intestinal wall thickening is the main manifestation of CD on CT, and the thickness is usually within 15 mm. In the acute stage, the intestinal wall can show the stratification phenomenon, manifested as the target sign or double halo sign, the low-density ring is caused by submucosal tissue edema, and the mucosa and serous membrane in the inflammatory activity stage can be strengthened during enhanced scanning. Intestinal stenosis can be seen in chronic stage. Localized lymph nodes in the mesentery are enlarged, usually in the range of 3 to 8 mm; on enhanced scan, the mesenteric blood vessels are thickened and twisted, the small straight arteries are elongated, widened in separation, and arranged in a comb-like pattern along the intestinal wall, known as the comb sign, which often indicates that the CD is in the active stage. When a fistula is formed, the fistula is seen to contain gas or contrast on CT.^[5]^

CD should be considered clinically in those with chronic onset, recurrent right lower abdominal or periumbilical pain, diarrhea, and weight loss, especially when accompanied by intestinal obstruction, abdominal tenderness, abdominal mass, intestinal fistula, perianal lesions, and fever. The World Health Organization has proposed 6 diagnostic points for the diagnosis of CD, see Table 1, which has recently been recommended again by the World Gastroenterology Organization.^[6]^ For the newly diagnosed atypical cases, the diagnosis should be clarified gradually through follow-up observation.

**Table 1:** Diagnostic criteria for Crohn’s disease recommended by the World Health Organization.

Items	Clinical manifestation	Radiological imaging	Endoscopy	Biopsy	Surgical specimen
① Discontinuous or segmental changes	‐	+	+	‐	+
② Cobblestone-like appearance or longitudinal ulcers	‐	+	+	‐	+
③ Whole-wall inflammatory response changes	+	+	‐	+	+
④ Non-caseous granulomas	‐	‐	‐	+	+
⑤ Fissures, fistulas	+	+	‐	‐	+
⑥ Perianal lesions	+	‐	‐	‐	‐

Patients with ①, ②, ③ are suspected; add ④, ⑤, ⑥ one of the 3 can be diagnosed; you can also be diagnosed with ④, as long as you add ①, ②, ③ 2 of the 3. “‐” means that there is no such manifestation, “+” means that there is such manifestation.

CD needs to be distinguished from various intestinal infectious diseases. Due to the high incidence of various pathogens in China, diarrhea <6 weeks needs to be differentiated from most infectious enteritis.^[7]^ In the context of this group, CD and bacillary dysentery have the following differences. ① Acute bacillary dysentery is distinctly seasonal, occurring most often in summer and fall, whereas CD is not; most patients with acute bacillary dysentery tend to have a history of unclean diet, whereas patients with CD do not have a history of unclean diet. ② Acute bacillary dysentery onset of acute, high fever with chills, the course of the disease is generally 1 to 2 months; CD is generally low or moderate fever, a few flaccid hyperthermia. ③ In physical examination, patients with acute bacillary dysentery generally have no anemia appearance, obvious left lower abdominal tenderness, no rebound pain, and no mass; patients with CD have anemic appearance, usually periumbilical and lower abdominal tenderness, mostly on the right side, and some patients can feel the abdominal mass. ④ In laboratory examination, blood tests in patients with bacillary dysentery have elevated leukocytes and neutrophils; anemia is common in CD. Fecal cultures are positive for bacillary dysentery and negative for CD. ⑤ Acute bacillary dysentery is effectively treated with effective antibiotics, while CD is not. ⑥ The diagnosis can be confirmed by endoscopy and pathological examination. Bacillary dysentery is characterized by diffuse hyperemia, edema and superficial ulcers of the intestinal mucosa, mainly the rectum and sigmoid colon. CD lesions are segmentally distributed, with longitudinal ulcers, pebble-like mucosa, normal interlesion mucosa and rare rectal involvement.

Analysis of the causes of easy misdiagnosis. (1) There is a lack of recognition that a wide range of diseases can cause pus and blood stools as a clinical manifestation. When any pathogenic bacteria and/or toxins of the intestine and their physicochemical factors act on the intestinal wall, the inflammatory damage of the intestinal mucosa can cause congestion and edema, resulting in water absorption dysfunction, and the increase of water in the intestinal tract will cause diarrhea or loose stools. When the inflammation spreads to the mucous lamina propria, it can cause circulation obstruction of small blood vessels, leading to local ischemia and hypoxia, resulting in degeneration and necrosis of mucosal epithelial cells, which will form ulcers and produce pus and blood stools after the necrosis falls off. Mucous stool or mucous blood stool or pus blood stool are not specific symptom of bacillary dysentery, we should pay attention to other clinical manifestations, understand the personality and characteristics of various diseases, so as to reduce misdiagnosis. (2) Phagocytes are not specific cells for the diagnosis of bacillary dysentery. Red, white, and phagocytic cells are often present in bacillary dysentery, but phagocytes are also not specific for bacillary dysentery. Phagocytes are often found in the intestines that are infected by a variety of factors such as bacteria, viruses, protozoa, etc., as well as where there are necrotic cells in the intestines. Any intestinal inflammation or mechanical irritation can lead to leukocytosis and phagocytosis in the stool, so the diagnosis of bacillary dysentery cannot be solely based on stool routine or fecal phagocyte detection.

## 4. Conclusion

Lessons to be learned from this patient. ① In order to reduce misdiagnosis, we should first pay attention to the detailed medical history and careful physical examination. ② The results of auxiliary examinations and tests should be treated correctly. Overreliance on auxiliary examination and one-sided emphasis on a certain test result are also important factors leading to misdiagnosis. ③ Anal examination and endoscopy should be emphasized and made routine in patients with chronic diarrhea. ④ The effectiveness of treatment is the best test of a correct diagnosis. Patients initially diagnosed with “bacillary dysentery” should be alerted to the presence of other intestinal diseases if antibiotic treatment is ineffective. ⑤ Closely observe the changes of the disease and analyze the clinical data comprehensively. Certain patients have atypical or diverse clinical manifestations, so patients should be observed after admission for a comprehensive analysis of the changes in the condition, in order to timely modify the diagnosis and treatment plan to avoid delays in diagnosis and treatment.

## Author contributions

**Conceptualization:** Xi Wang, Wen Ming.

**Formal analysis:** Xi Wang, Wen Ming, Guobin He.

**Investigation:** Hui Chen, Jingtong Tang.

**Methodology:** Xi Wang, Guobin He.

**Project administration:** Xi Wang, Guobin He.

**Resources:** Xi Wang, Wen Ming.

**Supervision:** Guobin He.

**Validation:** Wen Ming.

**Writing – original draft:** Xi Wang.

**Writing – review & editing:** Xi Wang, Wen Ming, Guobin He.
